# Ammonium Nitrogen Tolerant *Chlorella* Strain Screening and Its Damaging Effects on Photosynthesis

**DOI:** 10.3389/fmicb.2018.03250

**Published:** 2019-01-07

**Authors:** Jie Wang, Wei Zhou, Hui Chen, Jiao Zhan, Chenliu He, Qiang Wang

**Affiliations:** ^1^Key Laboratory of Algal Biology, Institute of Hydrobiology, Chinese Academy of Sciences, Wuhan, China; ^2^University of the Chinese Academy of Sciences, Beijing, China; ^3^Donghu Experimental Station of Lake Ecosystems, State Key Laboratory of Freshwater Ecology and Biotechnology of China, Institute of Hydrobiology, Chinese Academy of Sciences, Wuhan, China

**Keywords:** ammonium nitrogen, biological remediation, *Chlorella*, oxygen-evolving complex, photosynthesis

## Abstract

Nitrogen is an essential nutrient element. Ammonium nitrogen, one of the most common nitrogen sources, is found in various habitats, especially wastewater. However, excessive amounts of ammonium nitrogen can be toxic to phytoplankton, higher plants, fish, and other animals, and microorganisms. In this study, we explored the tolerance of green algae to ammonium nitrogen using 10 *Chlorella* strains. High concentrations of ammonium nitrogen directly inhibited the growth of *Chlorella*, but the degree of inhibition varied by strain. With the EC_50_ of 1.6 and 0.4 g L^−1^, FACHB-1563 and FACHB-1216, respectively had the highest and lowest tolerance to ammonium nitrogen among all strains tested, suggesting that FACHB-1563 could potentially be used to remove excess ammonium nitrogen from wastewater in bioremediation efforts. Two strains with the highest and lowest tolerance to ammonium nitrogen were selected to further explore the inhibitory effect of ammonium nitrogen on *Chlorella*. Analysis of chlorophyll fluorescence, oxygen evolution, and photosynthesis proteins via immunoblot showed that photosystem II (PSII) had been damaged when exposed to high levels of ammonium nitrogen, with the oxygen-evolving complex as the primary site, and electron transport from ${\text{Q}}_{\text{A}}^{-}$ to Q_B_ was subsequently inhibited by this treatment. A working model of ammonium nitrogen competition between N assimilation and PSII damage is proposed to elucidate that the assimilation rate of ammonium nitrogen by algae strains determines the tolerance of cells to ammonium nitrogen toxicity.

## Introduction

Nitrogen is one of the most important elements on Earth. Many different forms of nitrogen exist in the biosphere. Molecular nitrogen (N_2_) is the main form, accounting for ~78% of the Earth's atmosphere. Free nitrogen molecules in the atmosphere can be converted into combined nitrogen via atmospheric, industrial, and biological nitrogen fixation (Lanyon, 1995; Nishibayashi et al., 2004; Olivares et al., 2013). Combined nitrogen, primarily ammonium nitrogen and nitrate nitrogen, subsequently become the objects of intense competition among plants and microorganisms (Costa et al., 2001; Alves et al., 2016). Ammonium nitrogen can be directly assimilated into amino acids via the enzymes glutamine synthetase (GS) and glutamate synthase (GOGAT) (Wu et al., 2016), whereas nitrate nitrogen must first be reduced to nitrite nitrogen in the cytosol, after which it is immediately reduced to ammonium nitrogen in chloroplasts or plastids (Monier et al., 2015). Nitrate reductase and nitrite reductase catalyze these reduction reactions, respectively (Daniel-Vedele et al., 1998; Fernandez and Galvan, 2007). An additional reduction process, nitrate nitrogen assimilation, requires more energy, and ammonium nitrogen is considered to be more efficient than nitrate nitrogen in terms of energy utilization (Ruan and Giordano, 2017). However, ammonium nitrogen can be toxic to many organisms, particularly plants, and oxygenic photosynthetic microorganisms (Drath et al., 2008; Markou et al., 2014).

Ammonium nitrogen, which usually refers to nitrogen in the form of free ammoniacal nitrogen (NH_3_) and ammonium ions (N${\text{H}}_{4}^{+}$), is present in natural waters, with higher levels found in wastewater, such as domestic wastewater (Cruz et al., 2018), industrial waste (Huang et al., 2009), and aquaculture waste (Lu et al., 2015). NH_3_ and N${\text{H}}_{4}^{+}$ can be present in a solution simultaneously. The pKa (ion dissociation constant) of the N${\text{H}}_{4}^{+}$/NH_3_ buffer system is about 9.25 at 25°C, meaning that at pH < 9.25, the dominant form is N${\text{H}}_{4}^{+}$, while at pH > 9.25, the dominant form is NH_3_. The content of NH_3_ in a specific concentration of ammonium nitrogen can be calculated using the equation NH_3_ (%) = 100/(1 + 10 ^*^ (pKa – pH)) (Körner et al., 2001), which is closely related to the pH of the medium and increases with increasing pH.

Several mechanisms have been proposed to explain the toxic effects of ammonium nitrogen: the ammonium nitrogen assimilation process destroys the carbon and nitrogen balance in plants (Kronzucker et al., 1998); ammonium nitrogen transport disrupts intracellular pH balance (Pearson and Stewart, 1993); the long-term use of N${\text{H}}_{4}^{+}$ leads to the loss of cations such as Mg^2+^, Ca^2+^, and K^+^ in the cell, leading to a nutrient imbalance (Li et al., 2012); the ineffective transmembrane cycling of NH_3_ leads to energy loss (Speer and Kaiser, 1994); and NH_3_ affects the oxygen-evolving complex (OEC) by displacing a water ligand to the outer Mn cluster of the OEC (Dai et al., 2008; Tsuno et al., 2011). However, to what extent phytoplankton can tolerate and/or efficiently assimilate ammonium nitrogen is unknown, and the primary target of ammonium nitrogen damage in the photosynthetic machinery remains to be identified.

The green alga *Chlorella* is highly resistant to ammonium nitrogen. *Chlorella* can use ammonium nitrogen for growth, making it possible to use this alga for bioremediation to remove ammonium nitrogen (Tam and Wong, 1996). In addition, due to the abundant proteins and biolipids found in *Chlorella*, it is sometimes used as a health product or biological bait, as well as for biodiesel production (Zhang et al., 2014).

In this study, we screened 10 *Chlorella* strains for tolerance to ammonium nitrogen, analyzed the underlying tolerance mechanism, and proposed a working model based on the competition between N assimilation and PSII damage by ammonium nitrogen in the chloroplast.

## Materials and Methods

### Algae Strains

*Chlorella* strains *Chlorella sorokiniana* W1, *Chlorella* sp. W2, *C. sorokiniana* W3, *C. sorokiniana* W4, *C. sorokiniana* W5, and *C. sorokiniana* W6 were collected from the wild and preserved in the China Center for Type Culture Collection (Chen et al., 2017). Strains FACHB-1, FACHB-1216, FACHB-1535, and FACHB-1563 were obtained from the Freshwater Algae Culture Collection of the Institute of Hydrobiology, Chinese Academy of Sciences (Li et al., 2016).

### Growth Conditions and Ammonium Nitrogen Treatment

The N-sufficient medium used for the control group was full-strength BG11 medium, whereas N-deficient BG11 medium supplied with high concentration NH_4_Cl (HC, 0.5 g L^−1^) or low concentration NH_4_Cl (LC, 0.05 g L^−1^) was used for the treatment groups. The initial pH of the medium for both groups was adjusted to ~7.5 by adding NaOH or HCl; at this pH, N${\text{H}}_{4}^{+}$ is the dominant form of ammonium nitrogen, whereas NH_3_ comprises only ~2.5% of the total. *Chlorella* cells at the mid-logarithmic phase (OD_700_ ~0.8) were harvested by centrifugation at 3,000 g for 3 min at 25°C. The pellet was washed with N-free BG11 medium and re-suspended to OD_700_ 0.1 for both the control and treatment groups. All *Chlorella* strains were cultured in 100 mL Erlenmeyer flasks containing 50 mL of culture medium at 25°C with continuous illumination at 70 μmol m^−2^ s^−1^ and continuous rocking on a shaker at 150 rpm. The OD_700_ value was measured daily with a spectrophotometer, and the data were used to track cell growth. The pH and ammonium nitrogen content of the supernatant were also measured daily. The pH was measured with a glass electrode, and the ammonium nitrogen content was measured as described previously (Tam and Wong, 1996).

### NH_3_ Sensitivity and Adaptation Analysis of Various *Chlorella* Strains

*Chlorella* strains grown in N-deficient BG11 medium supplied with different concentrations of NH_4_Cl (0.1, 0.5, 1, 1.5, 2, 2.5, 3, and 4 g L^−1^) served as the treatment groups, and cells grown in full-strength BG11 medium served as the control group. All *Chlorella* strains were grown under continuous illumination at 70 μmol m^−2^ s^−1^ (white light) with the same initial OD_700_ of 0.4. To increase the proportion of NH_3_ in ammonium nitrogen, the initial pH level of the media for both the control and treatment groups was adjusted to 9.25 by adding NaOH or HCl, at which point the content of NH_3_ theoretically occupies ~56% of the total. The Fv/Fm of each sample was measured after 2 h of treatment. The 50% effective concentration (EC_50_), corresponding to the NH_4_Cl concentration at which the Fv/Fm is half that of the control, was used to reflect the relative level of ammonium nitrogen tolerance (Dai et al., 2014). The EC_50_ was calculated by Probit analysis in the SPSS-19 (Hoekstra, 1991).

The *Chlorella* strains with the highest and lowest tolerance to ammonium nitrogen (FACHB-1563 and FACHB-1216) were chosen among the 10 *Chlorella* strains. The mean value of the EC_50_ (1 g L^−1^) of the two selected *Chlorella* strains was used as the NH_4_Cl treatment concentration in subsequent analyses. The growth conditions were the same as those used for screening.

### GOGAT and GS Activity Measurement

GS and GOGAT activity were measured according to Martin-Figueroa et al. (2000) using GS and GOGAT test kits purchased from the Beijing Solarbio Science & Technology Co., Ltd., China.

### Chlorophyll Fluorescence Measurements

Quantum Yield (QY), chlorophyll fluorescence induction kinetics (OJIP), and Non-Photochemical Quenching (NPQ) were measured using an AquaPen-C AP-C 100 fluorometer. Red light was used as the measuring light, the measuring flash pulse was set to 0.009 μmol m^−2^ s^−1^, the saturating pulse was 2,100 μmol m^−2^ s^−1^, and the actinic light (A-pulse) level was 400 μmol m^−2^ s^−1^ (Zhang et al., 2016). Chlorophyll fluorescence parameters Fv/Fm, Y(II), and Y(NO) and JIP-test parameters Qp and Mo, Ψo, φEo, and Wk were used in this study, which were calculated according to the following equations:

(1) $$\begin{array}{rcl} \text{Fv}/\text{Fm} & = & \left(\text{Fm}-\text{Fo}\right)/\text{Fm} \end{array}$$

(2) $$\begin{array}{rcl} \text{Y}\left(\text{II}\right) & = & \left(\text{Fm}-\text{F}\right)/\text{Fm} \end{array}$$

(3) $$\begin{array}{rcl} \text{Y}\left(\text{NO}\right) & = & \text{F}/\text{Fm} \end{array}$$

(4) $$\begin{array}{rcl} \text{Qp} & = & \left(\text{Fm}-\text{F}\right)/\left(\text{Fm}-\text{Fo}\right) \end{array}$$

(5) $$\begin{array}{rcl} \varphi \text{Eo} & = & \left(1-\left({\text{F}}_{\text{O}}/{\text{F}}_{\text{M}}\right)\right)*\text{Ψo} \end{array}$$

(6) $$\begin{array}{rcl} \text{Wk} & = & \left(\text{Ft}-\text{Fo}\right)/\left({\text{F}}_{\text{J}}-\text{Fo}\right) \end{array}$$

(7) $$\begin{array}{rcl} \text{Mo} & = & 4\left({\text{F}}_{300\mu \text{s}}-\text{Fo}\right)/\left({\text{F}}_{\text{M}}-\text{Fo}\right) \end{array}$$

(8) $$\begin{array}{rcl} \text{Ψo} & = & {\text{ETo}/\text{TRo}=1-\text{V}}_{\text{J}} \end{array}$$

### Photosynthetic Oxygen Evolution and Dark Respiration Rates

Photosynthetic oxygen evolution and dark respiration rates were measured as described by Zhang et al. (2013).

### SDS-PAGE and Immunoblot Analysis

SDS-PAGE and immunoblot analysis of cellular proteins were performed according to Zhang et al. (2016).

### Statistical Analysis

For each result shown, the data are the average of three biological replicates. Data were analyzed using SPSS-19. Significance was determined using a one-way ANOVA at the *P* < 0.05 or *P* < 0.01 confidence limits.

## Results

### Ammonium Nitrogen Directly or Indirectly Inhibits *Chlorella* Growth

**Figure 1:**
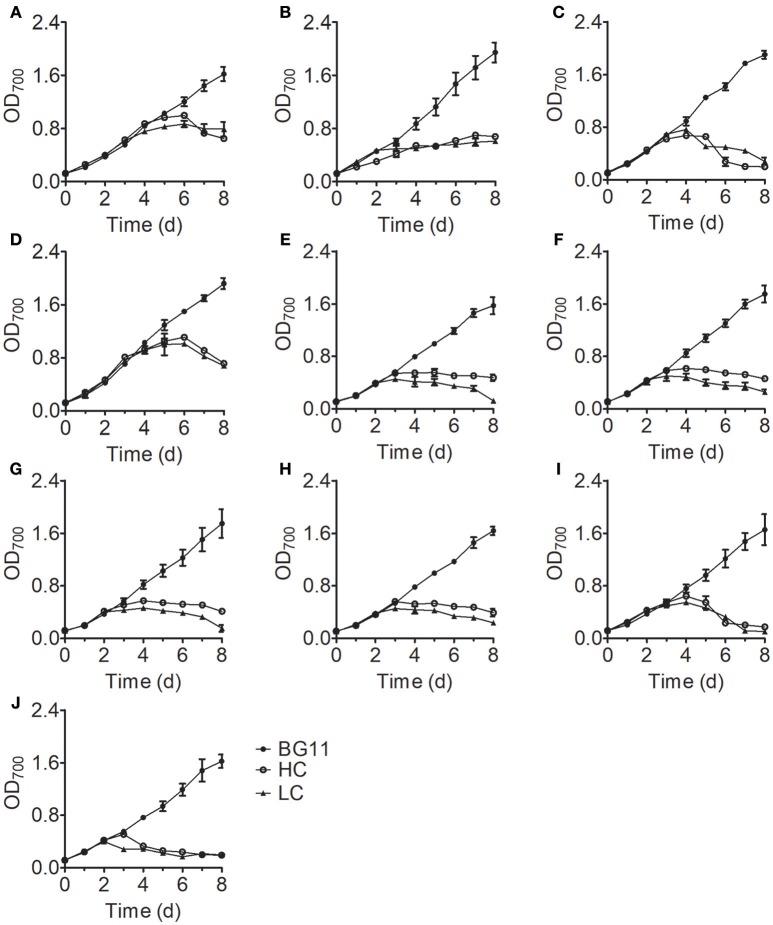
Growth curves of 10 *Chlorella* strains cultivated in BG11 medium (BG11) and in N-deficient BG11 medium supplemented with 0.5 g L^−1^ NH_4_Cl (HC) or 0.05 g L^−1^ NH_4_Cl (LC). **(A–J)** Represent *Chlorella* strain FACHB-1, FACHB-1216, FACHB-1535, FACHB-1563, W1, W2, W3, W4, W5, and W6, respectively. For all figures, the data points represent the means of three replicate experiments for each independent culture, with the SD of the means.

**Figure 2:**
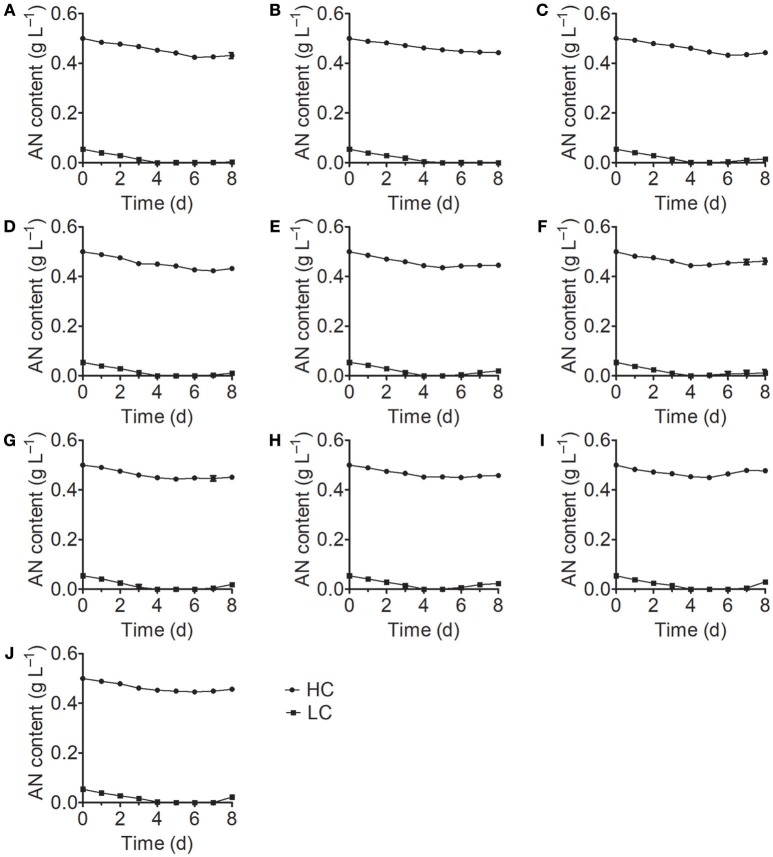
Ammonium nitrogen (AN) content of 10 *Chlorella* strains cultivated in HC (circles) and LC (squares) medium. **(A–J)** Represent *Chlorella* strain FACHB-1, FACHB-1216, FACHB-1535, FACHB-1563, W1, W2, W3, W4, W5, and W6, respectively.

Analysis of the growth curves of the 10 *Chlorella* strains under LC conditions revealed no significant differences (*P* > 0.05) compared to the control group cultured in standard BG11 medium for the first 3 days, after which the growth rates began to decrease and some *Chlorella* strains perished (Figure 1). When cultivated under HC conditions, most *Chlorella* strains showed similar growth patterns to those cultivated under LC conditions, but the growth of FACHB-1216 was inhibited at the beginning of the treatment. We also measured the ammonium nitrogen content in the growth medium, which exhibited similar tendencies for all 10 *Chlorella* strains (Figure 2). The ammonium nitrogen content gradually decreased during growth, even approaching 0 on day 3 under LC conditions. By contrast, under HC conditions, only a slight decrease in ammonium nitrogen content (~10%) was detected. The residual ammonium nitrogen content was sufficiently high (0.45 g L^−1^) when cell growth under HC conditions was inhibited during the middle and later stages of growth. These results indicate that the algal growth inhibition or even death observed in the middle and late stages in the HC-treatment group was not due to the consumption of ammonium nitrogen.

**Figure 3:**
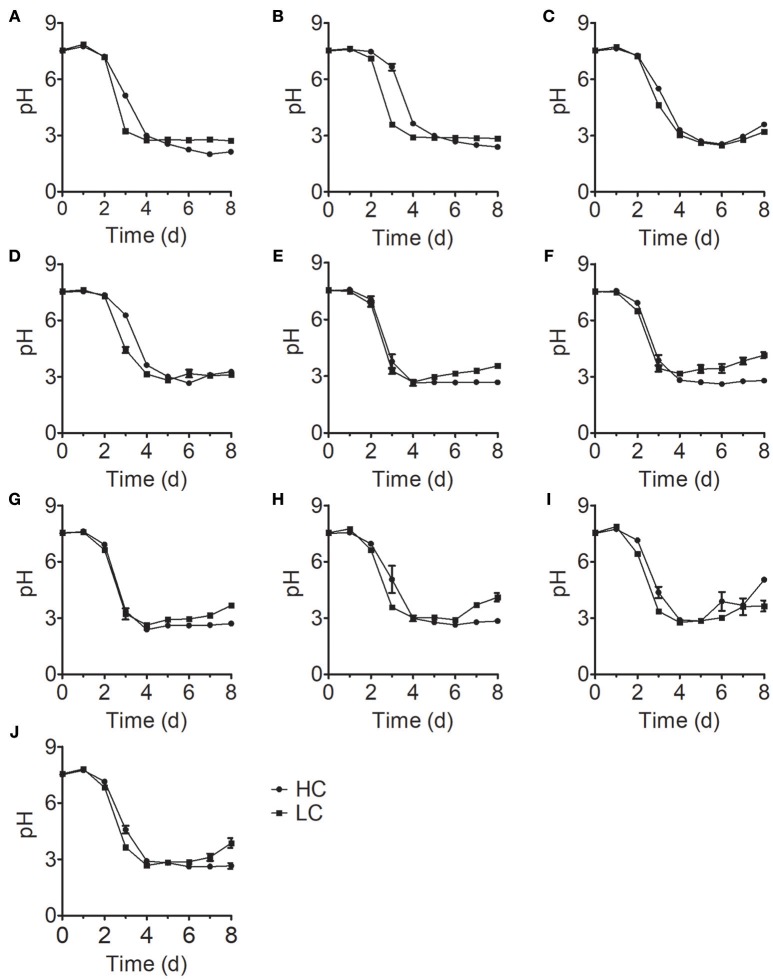
pH changes in 10 *Chlorella* strains cultivated in HC (circles) and LC (squares) medium. **(A–J)** Represent *Chlorella* strain FACHB-1, FACHB-1216, FACHB-1535, FACHB-1563, W1, W2, W3, W4, W5, and W6, respectively.

The initial pH value of the culture medium was ~7.5 for all treatments; at this pH, N${\text{H}}_{4}^{+}$ is the dominant form of ammonium nitrogen. During the early stage of growth, the pH values of all samples in the treatment group were stable. However, these values rapidly decreased to ~pH 3 on the fourth day of culture and stabilized with increasing culture time (Figure 3), which is consistent with the time of growth inhibition for most *Chlorella* strains (Figure 1). In acidic environment, many enzymes become inactive (Williams and Colman, 1996) and gross oxygen production was lowered (Ihnken et al., 2014) in *Chlorella*. Therefore, we speculated that the growth inhibition in the middle and late stages was due to acidification of the medium. However, we detected a significant difference in cell growth in FACHB-1216 between control and HC conditions throughout the experiment, suggesting that ammonium nitrogen can also directly inhibit *Chlorella* growth.

### The Ability to Assimilate Ammonium Nitrogen Determines the Tolerance of *Chlorella* to Ammonium Nitrogen

As the amount of free NH_3_ in a specific concentration of ammonium nitrogen solution is pH dependent, we increased the initial pH values of the culture medium to pH 9.25 to increase the content of NH_3_ and measured the EC_50_ of the 10 *Chlorella* strains to further explore the mechanism underlying tolerance to ammonium nitrogen (Table 1). Most *Chlorella* strains tested were highly tolerant to ammonium nitrogen (EC_50_ > 1 g L^−1^); even the EC_50_ values of FACHB-1563 and FACHB-1535 were >1.6 g L^−1^. FACHB-1216 showed the lowest tolerance to ammonium nitrogen, with an EC_50_ of 0.4 g L^−1^. The high sensitivity of FACHB-1216 to ammonium nitrogen might explain why cell growth in this culture was inhibited throughout the cultivation period (Figure 1).

**Table 1:** EC_50_ of 10 *Chlorella* strains after 2 h of ammonium nitrogen treatment.

***Chlorella* strain**	**95% confidence limit for concentration**		
	**Estimate (g L^**−1**^)**	**Lower limit (g L^**−1**^)**	**Upper limit (g L^**−1**^)**
FACHB-1	0.827^A^	0.646	1.024
FACHB-1216	0.404^B^	0.371	0.437
FACHB-1535	1.665^C^	1.570	1.765
FACHB-1563	1.679^C^	1.499	1.885
W1	0.906^A^	0.790	1.023
W2	1.193^A^	1.093	1.290
W3	1.159^A^	0.999	1.322
W4	1.185^A^	1.078	1.290
W5	1.135^A^	0.889	1.401
W6	0.930^A^	0.720	1.154

*Significance was determined using a one-way ANOVA. Different superscript letters (A, B, and C) indicate significant differences among strains (P < 0.05)*.

**Figure 4:**
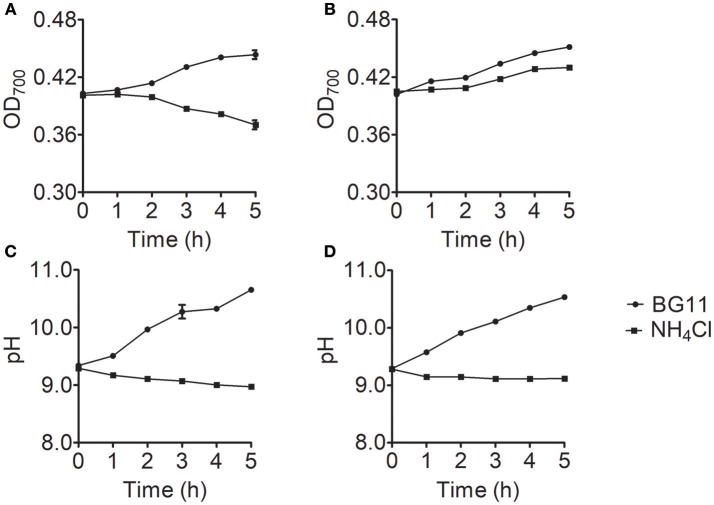
Growth and pH changes in *Chlorella* strain FACHB-1216 and FACHB-1563 cultured in BG11 (circles) and 1 g L^−1^ NH_4_Cl (squares). **(A,B)** Represent the growth of *Chlorella* strain FACHB-1216 and FACHB-1563, respectively; **(C,D)** Represent pH changes in *Chlorella* strain FACHB-1216 and FACHB-1563, respectively.

To identify the cause of the differences in ammonium nitrogen tolerance among strains, we selected the *Chlorella* strains with the highest and lowest tolerance to ammonium nitrogen (FACHB-1563 and FACHB-1216). The growth rate significantly differed (*P* < 0.05) between FACHB-1216 and FACHB-1563, whereas pH values exhibited similar trends (Figure 4), indicating that the tolerance of these lines to ammonium nitrogen indeed differed.

**Figure 5:**
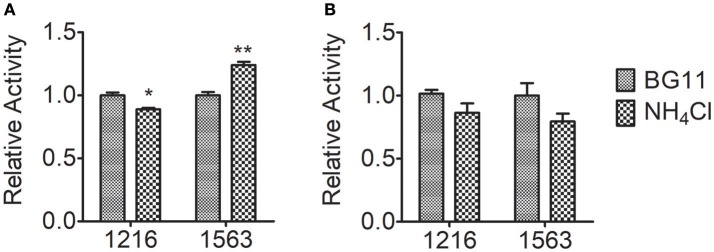
Relative enzyme activity of GS and GOGAT in *Chlorella* strain FACHB-1216 and FACHB-1563 after incubation for 5 h in BG11 and 1 g L^−1^ NH_4_Cl. **(A,B)** Represent the enzymes GS and GOGAT, respectively. The control (BG11) values were set to 1 to facilitate comparison. The significance of the differences between the control (BG11) and other values was tested using a one-way ANOVA. *P < 0.05; **P < 0.01.

In nitrogen metabolism, ammonium nitrogen are fixed via the glutamate–glutamine cycle, in which ammonium nitrogen combines with glutamate catalyzed by GS to form glutamine, and GOGAT transfers the amide group of glutamine to 2-oxoglutarate, yielding two molecules of glutamate, followed by re-distribution of the assimilated nitrogen into other key molecules, e.g., amino acids and nucleic acids (Chen et al., 2017). To further investigate the contribution of nitrogen assimilation to the tolerance of *Chlorella* to ammonium nitrogen, we measured the activity of the two key enzymes that catalyze ammonium nitrogen assimilation, GOGAT and GS (Figure 5). After 5 h of NH_4_Cl treatment, GOGAT activity in both FACHB-1216 and FACHB-1563 decreased slightly. Notably, during this period, GS activity in FACHB-1216 decreased (*P* < 0.05), but GS activity in FACHB-1563 increased significantly (*P* < 0.01), suggesting that the increase in GS activity in this strain promoted ammonium nitrogen assimilation and alleviated the toxicity of this compound to the cell, thereby leading to better ammonium nitrogen tolerance in FACHB-1563 compared to FACHB-1216.

Thus, for the strain with high sensitivity to ammonium nitrogen, ammonium nitrogen had a direct toxic effect on the algae. For the strain with high tolerance to ammonium nitrogen, cell growth was not affected at the initial stages of growth. However, the culture medium gradually became acidified with the constant assimilation of N${\text{H}}_{4}^{+}$, which indirectly led to growth inhibition, and even death of the algae. Therefore, the ability of *Chlorella* to assimilate ammonium nitrogen determines its tolerance to this compound.

### High Levels of Ammonium Nitrogen Damage PSII in *Chlorella*

**Figure 6:**
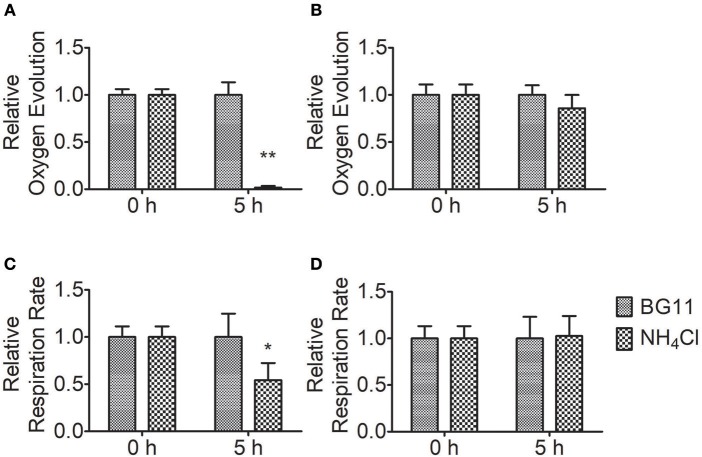
Relative photosynthetic oxygen evolution rate and respiratory oxygen consumption of *Chlorella* strains FACHB-1216 and FACHB-1563 cultured in BG11 and 1 g L^−1^ NH_4_Cl. **(A,B)** Represent the photosynthetic oxygen evolution of *Chlorella* strain FACHB-1216 and FACHB-1563, respectively; **(C,D)** Represent the respiratory oxygen consumption of *Chlorella* strain FACHB-1216 and FACHB-1563, respectively. The significance of the differences between the same sample at 0 h and 5 h was tested using a one-way ANOVA. *P < 0.05; **P < 0.01.

To measure the effect of ammonium nitrogen on photosynthesis, we analyzed the oxygen evolution and respiration rates of FACHB-1216 and FACHB-1563 (Figure 6). Compared to the control, the oxygen evolution and respiration rates in FACHB-1563 were not markedly different (*P* > 0.05), whereas these values were markedly different in FACHB-1216 (*P* < 0.05). Oxygen evolution dropped to 0 and the respiration rate declined ~50% after 5 h of HC treatment in FACHB-1216. The results indicated that ammonium nitrogen has adverse effects on the photosynthesis, and the degrees were strain dependent.

**Figure 7:**
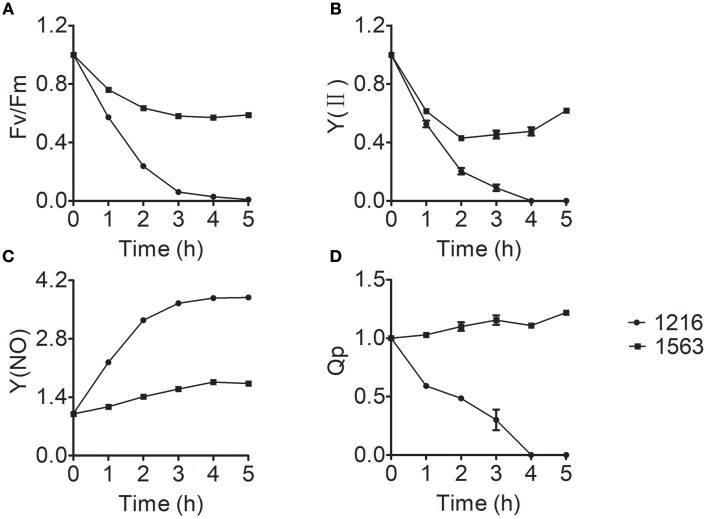
**(A–D)** Relative fluorescence parameters of *Chlorella* strain FACHB-1216 (circles) and FACHB-1563 (squares) cultured in BG11 and 1 g L^−1^ NH_4_Cl. The control (0 h) values were set to 1.

Chlorophyll fluorescence measurement, a simple, quick, and reliable method to study the changes of photosynthesis, has long been considered one of the most sensitive and noninvasive tools to investigate stress responses of photosynthesis under unfavorable conditions (Krause, 1991). To gain more insight into the effect of ammonium nitrogen on photosynthesis, various chlorophyll fluorescence parameters were investigated. The chlorophyll fluorescence parameters of maximum photosynthetic efficiency (Fv/Fm), practical photosynthetic efficiency [Y(II)], non-regulated energy dissipation [Y(NO)], and photochemical quenching (Qp) values of FACHB-1216 and FACHB-1563 were measured to further explored the effect of ammonium nitrogen on photosynthesis in this study. The decreased values of Fv/Fm and Y(II) suggested that both the capacity and activity of photosynthesis were negatively affected by the treatment, indicating that PSII is impaired (Chen et al., 2016), which could be further proved by the increased Y(NO) and decreased Qp. In FACHB-1563, Fv/Fm and Y(II) declined by ~40 and 50%, respectively, after a 5-h cultivation under HC conditions but dropped to 0 in FACHB-1216 (Figures 7A,B). In contrast to the slight increase in Y(NO) in FACHB-1563, this value increased nearly four-fold in FACHB-1216 (Figure 7C). The Qp value of FACHB-1563 was stable, whereas it dropped to 0 by the fourth hour of treatment in FACHB-1216 (Figure 7D).

**Figure 8:**
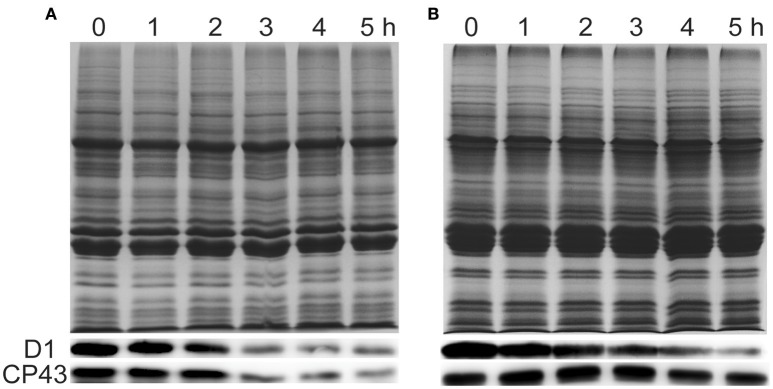
Immunoblot analysis was performed using an antibody specific to D1 and CP43 (lower panels). Equal loading was confirmed by Coomassie brilliant blue staining (upper panels). **(A,B)** Represent *Chlorella* strain FACHB-1216 and FACHB-1563, respectively.

To investigate whether the damage of PSII in response to ammonium nitrogen treatment is associated with changes in PSII protein content, we examined the effects of ammonium nitrogen on several major PSII proteins by immunoblot analysis (Figure 8). D1 protein content decreased in both FACHB-1216 and FACHB-1563 with increasing culture time. In FACHB-1216, CP43 protein levels showed the same tendency as D1, but in FACHB-1563, CP43 protein levels were stable, revealing more serious damage to PSII in FACHB-1216. Together, these results suggested that the toxicity of ammonium nitrogen to *Chlorella* is due to its damaging effects on PSII, especially in the highly sensitive strain, FACHB-1216.

### OEC Is the Primary Site of Damage by Ammonium Nitrogen

**Figure 9:**
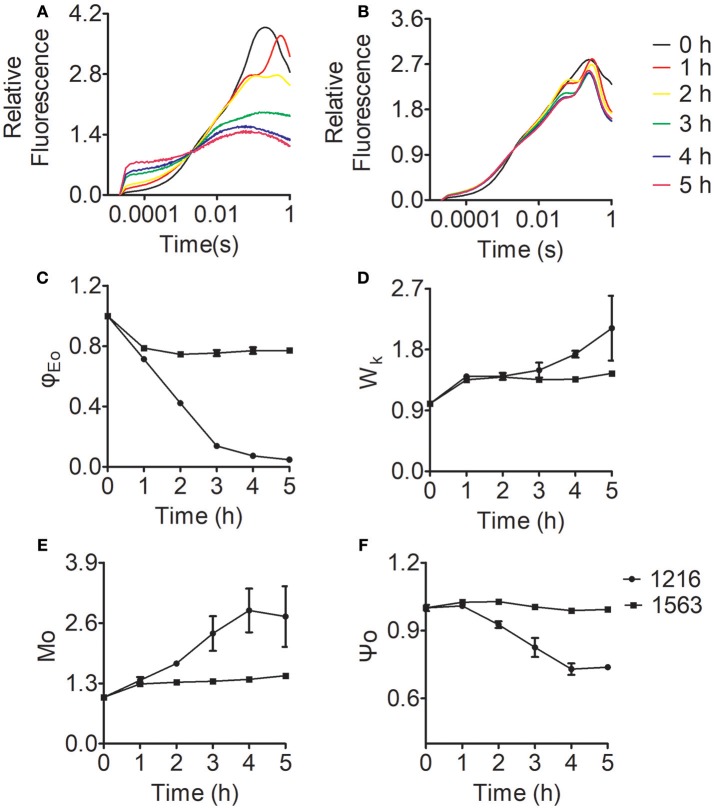
Relative variable fluorescence and JIP-test parameters of *Chlorella* FACHB-1216 and FACHB-1563 cultured in BG11 and 1 g L^−1^ NH_4_Cl. **(A,B)** Represent *Chlorella* strain FACHB-1216 and FACHB-1563, respectively. **(C–F)** Represent Mo, Ψo, ϕEo, and Wk, respectively. The control (0 h) values were set to 1.

To explore more details of the effects of ammonium nitrogen on PSII function in *Chlorella*, fast fluorescence kinetics, the OJIP curves for FACHB-1216 and FACHB-1563 were obtained and various parameters were calculated accordingly (Figure 9). As shown in Figure 9A, the curves of both FACHB-1216 and FACHB-1563 were elevated in the O-J segment within the first hour of HC treatment, and the K phase appeared, indicating that the OEC of PSII was damaged. The O-J segment continued to rise in FACHB-1216, but tended to be stable in FACHB-1563, which has high tolerance to ammonium nitrogen, after the first hour of treatment.

Among various JIP-test parameters, φEo represents the efficiency of the electron transport chain; decreasing values reflect inhibited electron transport in PSII (Strasser et al., 2004; Pan et al., 2008). φEo decreased in both FACHB-1216 and FACHB-1563 during the first hour of HC treatment, indicating that electron transfer was affected in both strains. The φEo value of FACHB-1216 continued to decrease, dropping to almost 0 at 5 h of treatment, whereas no significant changes were detected in FACHB-1563. These results indicate that electron transport was more severely inhibited in FACHB-1216 than in FACHB-1563 (Figure 9C).

Wk reflects the condition of the OEC, and increases with damaging OEC (Zhang et al., 2017). During the first hour of exposure to high ammonium nitrogen levels, Wk increased rapidly (by nearly 1.5-fold) in both FACHB-1216 and FACHB-1563, indicating initial damage to the OEC. Subsequently, the Wk value continued to increase (by two-fold) in FACHB-1216, but remained stable in FACHB-1563 (Figure 9D).

Mo is the initial slope of the OJIP curve, which represents the Q_A_ reduction rate; whereas the Ψo value reflects the probability that captured excitons will transfer electrons to ${\text{Q}}_{\text{A}}^{-}$, the downstream electron acceptor in the electron transport chain (Zhang et al., 2016). Therefore, changes in Mo and Ψo reflect changes in electron transport from ${\text{Q}}_{\text{A}}^{-}$ to Q_B_. In FACHB-1563, Mo and Ψo did not noticeably change throughout the culture period. By contrast, these values were stable in FACHB-1216 during the first hour of HC treatment, but subsequently Mo increased by nearly 300% and Ψo decreased by ~30% (Figures 9E,F). These results indicate that electron transport from ${\text{Q}}_{\text{A}}^{-}$ to Q_B_ was inhibited in FACHB-1216 after the first hour of treatment with high levels of ammonium nitrogen.

In summary, ammonium nitrogen inhibited the electron transport chain efficiency of PSII. OEC was the primary target site and was rapidly damaged. Electron transport from ${\text{Q}}_{\text{A}}^{-}$ to Q_B_ was also inhibited by ammonium nitrogen, but this inhibition followed damage to the OEC and could be alleviated in algae with high tolerance to ammonium nitrogen.

## Discussion

Ammonium nitrogen is one of the most energy-efficient nitrogen sources for algal metabolism (Ruan and Giordano, 2017). However, high concentrations of ammonium nitrogen are toxic and can inhibit algal growth (Gutierrez et al., 2016), which could be due to the effects of NH_3_ and N${\text{H}}_{4}^{+}$. NH_3_ is thought to be the most toxic form of ammonium nitrogen due it its lack of charge and its lipid solubility, allowing it to readily diffuse across the cell membrane (Collos and Harrison, 2014). N${\text{H}}_{4}^{+}$ is far less toxic than NH_3_ (Azov and Goldman, 1982; Kallqvista and Svenson, 2003), as the transport of N${\text{H}}_{4}^{+}$ from extracellular regions requires the participation of transporters. When CO_2_ is used as carbon source, the pH of the medium is depended on the CO_2_ assimilation rate. If the dissolution rate of CO_2_ is greater than that of assimilation, the continuous CO_2_ dissolution results in the accumulation of HC${\text{O}}_{3}^{-}$ and H^+^, so excessive CO_2_ penetration will lead to acidification of the medium. On the contrary, if the assimilation rate is high, the amount of CO_2_ assimilation is not enough to satisfy the need of carbon sources for cell and HC${\text{O}}_{3}^{-}$ would be assimilated as carbon source by the cell, which makes the K^+^/Na^+^ accumulated in the medium, causing alkalization of the medium. In this study, the cells were cultured in BG11 with shaking but not aerating, and NaHCO_3_ and NaNO_3_ were the main carbon and nitrogen source. The continuous consumption of N${\text{O}}_{3}^{-}$ and HC${\text{O}}_{3}^{-}$ could lead to Na^+^ accumulation, both of which cause medium alkalization (Figure 4). When ammonium nitrogen was used as nitrogen source, the assimilation of N${\text{H}}_{4}^{+}$ does acidify the medium, and the assimilation of HC${\text{O}}_{3}^{-}$ does alkalinize it. However, OH^−^ produced during the HC${\text{O}}_{3}^{-}$ assimilation in the cell could not neutralize the excess H^+^ produced by N${\text{H}}_{4}^{+}$ assimilation, which led to medium acidification (Figure 4). Some studies reported that with gaseous CO_2_ as carbon source, excess H^+^ or OH^−^ produced during the assimilation in the cell was mainly decided by nitrogen source and one N${\text{H}}_{4}^{+}$ assimilated by cell would produce at least one H^+^ in plant cell cytoplasm (Raven and Smith, 1976; Raven, 1986; Andrews et al., 2013). Excess H^+^ should be neutralized to maintain cytoplasmic pH, and net H^+^ efflux from cell was often greater than its pH-regulation capacity (Raven, 1986), acidifying the surrounded environment. Usually, it is known that ammonium nitrogen can result in uncoupling of electron transfer and proton pumping, consequently, cause an acidification in cells of *Chlorella*. Thus the transformation of N${\text{H}}_{4}^{+}$ is restricted in chloroplasts and further regulated by its assimilation rate (Flores and Herrero, 2015). Therefore, the assimilation rate of ammonium nitrogen determines the tolerance of algae strain. Normally, the ratio of N${\text{H}}_{4}^{+}$ to NH_3_ in the growth medium is mainly determined by pH, with increasing pH levels leading to increased NH_3_ content (Collos and Harrison, 2014); thus, the form and toxicity of ammonium nitrogen present in the medium are associated with pH (Markou et al., 2014; Tan et al., 2016).

Here, we examined the tolerance of 10 *Chlorella* strains to ammonium nitrogen, which could be divided into three categories (Table 1): good tolerance (FACHB-1563 and FACHB-1535), fair tolerance (FACHB-1, W1, W2, W3, W4, W5, and W6), and poor tolerance (FACHB-1216). The growth of these 10 *Chlorella* strains under HC culture conditions, in which N${\text{H}}_{4}^{+}$ is the dominant form of ammonium nitrogen, could roughly be divided into two modes (Figure 1): fast–slow growth mode, in which rapid growth in the early stage is followed by reduced growth in the middle and late stages (FACHB-1, 1535, 1563, W1, W2, W3, W4, W5, and W6) and slow growth mode, in which inhibited growth occurs throughout the culture period (FACHB-1216). Algae with a fast–slow growth mode had fair or good tolerance to ammonium nitrogen and grew normally during the early stage of growth. However, as N${\text{H}}_{4}^{+}$ became assimilated by the cell, the medium gradually became acidified (Figure 3), which indirectly inhibited the growth of the algae (Xin et al., 2010). FACHB-1216, the only alga with a slow growth mode, had poor tolerance to ammonium nitrogen. High levels of ammonium nitrogen had a direct effect on its cell growth, which was inhibited throughout the culture period. Therefore, high levels of ammonium nitrogen can directly affect the growth of algae with poor tolerance of this compound. By contrast, the growth of algae with high tolerance to ammonium nitrogen is not affected at the initial stage of growth, but the assimilation of N${\text{H}}_{4}^{+}$ by the algae leads to acidification of the medium, which indirectly inhibits algal growth.

Ammonium nitrogen toxicity may be universal, but the threshold at which symptoms of toxicity become manifested differs widely among algae. Collos and Harrison (2014) compared the effects of high ammonium nitrogen concentrations on the growth of six classes of microalgae, and found that Chlorophytes were significantly more tolerant to high ammonium nitrogen levels than were diatoms, prymnesiophytes, dinoflagellates, and raphidophytes. Within the same class of algae, different strains also have significantly different levels of tolerance to ammonium nitrogen. The sensitivity of five cyanobacterial strains to ammonium nitrogen toxicity is as follows: Ge–Xian–Mi > *Anabaena azotica* FACHB 118 > *Microcystis aeruginosa* FACHB 905 > *M. aeruginosa* FACHB 315 > *Synechococcus* FACHB 805 (Dai et al., 2008). In this study, we assessed the tolerance of 10 *Chlorella* strains to ammonium nitrogen (Table 1), and found that the degree of tolerance is strain-specific, with FACHB-1563 and FACHB-1216, respectively, having the highest and lowest tolerance to ammonium nitrogen among the 10 *Chlorella* strains tested. The activity of GS-GOGAT display a marked difference (Figure 5) in the two strains with different tolerance to ammonium nitrogen. In the strain with high GS-GOGAT activity, N${\text{H}}_{4}^{+}$ can be effectively converted into organic nitrogen, thus avoiding the accumulation of N${\text{H}}_{4}^{+}$ in cells and effectively reducing ammonia toxicity, which suggested that the ability to assimilate ammonium nitrogen determines the tolerance of *Chlorella* to ammonium nitrogen. Same conclusion has also been drew by Gumenyuk (2003), and he found that the green algae with higher tolerance to high ammonium have higher GS/GDH activities. Remove of toxic nitrogen promptly would improve the tolerance of cell to nitrogen (Collos and Harrison, 2014).

Numerous studies have focused on screening microalgae for high tolerance to harmful substances to identify strains that can be used for the bioremediation of wastewater. *Monoraphidium* spp. SDEC-17, which can endure high-ammoniacal nitrogen conditions (>170 mg L^−1^), represents a promising candidate for algal biomass production and chemical energy recovery from complex wastewater (Jiang et al., 2016). However, besides high tolerance to ammonium nitrogen, both high nitrogen utilization efficiency and high growth rate are also deciding factors in algal bioremediation of ammonium nitrogen from wastewater. Paskuliakova et al. (2016) used four chlorophyte strains to reduce the level of ammonium nitrogen in landfill leachate and a reduction rate of 3.77 mg L^−1^ d^−1^ of ammonium nitrogen was detected in ~100 mg L^−1^ ammonium nitrogen. In the study of Tam and Wong (1996), *Chlorella vulgaris* was used to remove ammonium nitrogen, and a 3.61 mg L^−1^ d^−1^ removal efficiency was acquired in ~125 mg L^−1^ N${\text{H}}_{4}^{+}$-N. In this study, FACHB-1563, cultured in HC condition (500 mg L^−1^), showed the same growth rate with the control group in the first 3 days (Figure 1), and an ammonium nitrogen assimilation rate of 4.27 mg L^−1^ d^−1^ was observed with traces initial inoculation (OD_700_ 0.1) (Figure 2). Therefore, FACHB-1563 exhibits high tolerance to ammonium nitrogen (Table 1), with high removal rate (Figure 2) and high growth rate (Figure 1), making it an excellent candidate for use in removing ammonium nitrogen from wastewater.

Ammonium nitrogen is toxic to algae due to its damaging effects on photosynthesis (Azov and Goldman, 1982; Drath et al., 2008). Ammonium nitrogen directly induces photodamage to PSII rather than affecting the repair of photodamaged PSII (Dai et al., 2014). The toxic effects of ammonium nitrogen on photosynthesis appear to be complex, as this compound affects not only PSII, but also PSI, the electron transport chain, and the OEC (Markou et al., 2016). The OEC is the main site of damage, as NH_3_ is a structural analog of the substrate H_2_O and an inhibitor of the water oxidation reaction in PSII, and is thus able to replace substrate water molecules in the OEC in PSII (Hou et al., 2011; Tsuno et al., 2011). In this study, we explored the effect of ammonium nitrogen on PSII in algae based on an analysis of chlorophyll fluorescence, photosynthetic oxygen evolution, and photosynthetic protein detection (Figures 6–8). All of these parameters showed that PSII was negatively affected by ammonium nitrogen. Further fast kinetics OJIP analysis (Figure 9) revealed that the OEC was the first site damaged by ammonium nitrogen, followed by the electron transport from ${\text{Q}}_{\text{A}}^{-}$ to Q_B_.

**Figure 10:**
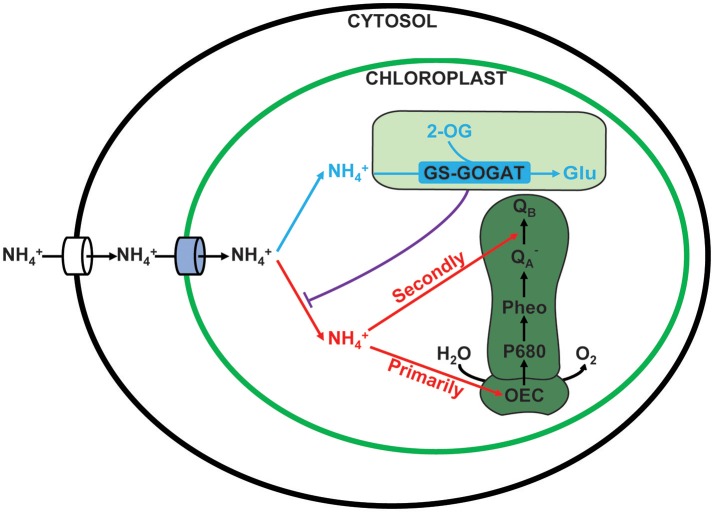
Competition between assimilation and PSII damage by ammonium nitrogen in the chloroplast. When ammonium nitrogen was transported into the chloroplast, it could be taken as: 1. N source and being assimilated into nontoxic organic nitrogen by GS-GOGAT (in blue), and 2. hazardous material that being toxic to photosynthesis, which initially damaging the OEC and then blocking electron transport from ${\text{Q}}_{\text{A}}^{-}$ to Q_B_ (in red). The damaging effects on PSII are dependent on the assimilation rate, which is further up to the activities of the GS-GOGAT.

Based on the current study, a working model of ammonium nitrogen competition between N assimilation and PSII damage was proposed (Figure 10): when transported into the chloroplast, N${\text{H}}_{4}^{+}$ can serve as both N source and hazardous material, thus could either be assimilated into non-toxic organic nitrogen (L-Glu) by GS-GOGAT, or being toxic to photosynthesis, which initially damaging the OEC and then blocking electron transport from ${\text{Q}}_{\text{A}}^{-}$ to Q_B_, and the GS-GOGAT catalyzed N${\text{H}}_{4}^{+}$ assimilation is a relief to its damaging effects on PSII. For algae strains with high GS-GOGAT activities, the toxic N${\text{H}}_{4}^{+}$ could be timely removed and transformed to avoid the immediate impact on PSII (Figures 6–9), thus showing high tolerance.

## Author Contributions

JW and QW designed the study. JW, WZ, HC, JZ, CH, and QW collected, analyzed, and interpreted the data. JW, HC, and QW wrote the manuscript.

### Conflict of Interest Statement

The authors declare that the research was conducted in the absence of any commercial or financial relationships that could be construed as a potential conflict of interest.
